# Rare Case of Asymptomatic Sternal Wire Migration Into the Ascending Thoracic Aorta Following Thoracic Surgery

**DOI:** 10.7759/cureus.89948

**Published:** 2025-08-12

**Authors:** François Reul, Valérie Lacroix

**Affiliations:** 1 Cardiovascular and Thoracic Surgery Department, Cliniques Universitaires Saint-Luc, Brussels, BEL

**Keywords:** ascending thoracic aorta, sternal closure, sternal wire, sternotomy complications, thoracic surgery

## Abstract

We report the case of a 62-year-old woman who underwent complex thoracic surgery for a Pancoast tumor, involving both posterior and anterior approaches, including a posterior cervical incision and a right anterior cervico-sterno-thoracotomy. Approximately one year after surgery, computed tomography angiography revealed a rare finding of a sternal wire embedded in the ascending aorta, posterior to the sternum. The patient was asymptomatic at the time of discovery. We present key imaging findings and describe the successful surgical re-intervention performed to remove the wire. This case highlights the critical importance of proper positioning and secure tightening of sternal wires during closure to prevent potentially serious complications in both cardiac and thoracic surgeries.

## Introduction

Median sternotomy with sternal wire closure is a well-established and widely used technique in cardiothoracic surgery [1]. Although generally safe, sternal wire-related complications can occur and may lead to serious consequences when adjacent mediastinal structures are involved. Reported cases often describe fractured or migrated wires causing pseudoaneurysms, cardiac tamponade, or vascular injury, typically requiring urgent intervention [2,3].

Migration of sternal wires into the ascending thoracic aorta is a rare complication. To the best of our knowledge, we report the first documented case of an unfractured sternal wire that progressively penetrated the wall of the ascending aorta, without symptoms or pseudoaneurysm formation, incidentally discovered during follow-up imaging.

## Case presentation

A 62-year-old woman underwent surgery for a Pancoast-Tobias squamous cell carcinoma (stage IIIA, pT4pN0cM0) of the right upper lung lobe, invading the posterior chest wall, cervical spine, and brachial plexus (C8-T1). The surgical procedure consisted of two stages: a posterior cervical approach for cervical spine intervention, followed by a Dartevelle-Grunenwald approach with a right anterior cervico-sterno-thoracotomy at the second intercostal space.

The immediate postoperative course was uneventful, featuring close analgesic monitoring, drainage of a right pleural effusion, and gradual improvement of right brachial paresis. The patient subsequently received adjuvant chemotherapy.

**Figure 1:**
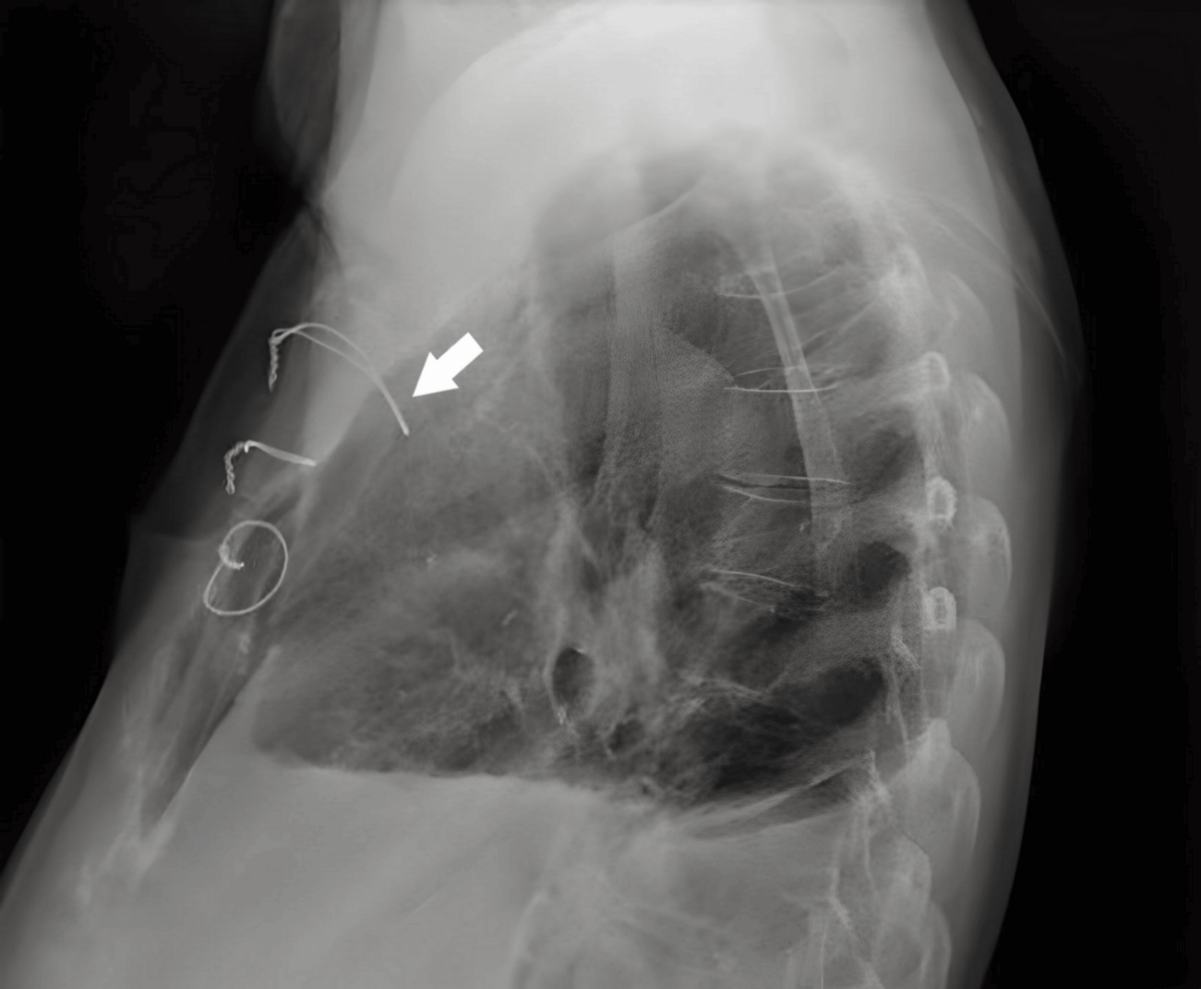
Chest X-ray showing protrusion of the loop of the first sternal wire into the anterior mediastinum

During follow-up, chest X-rays revealed a protrusion of the first sternal wire loop into the anterior mediastinum (Figure 1), a finding noted since the early postoperative days.

**Figure 2:**
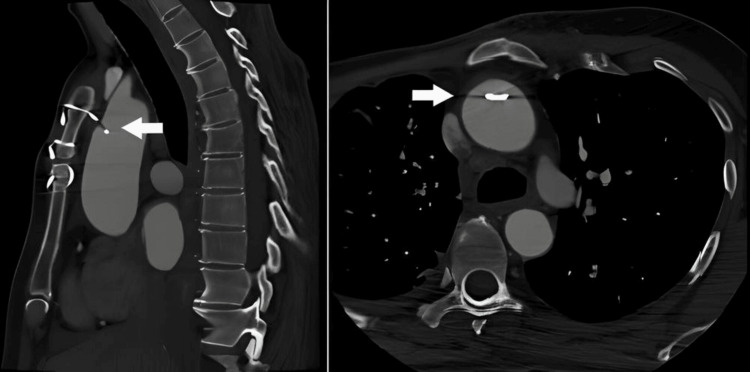
Computed tomography angiography showing a sternal wire penetrating the ascending thoracic aorta

Computed tomography angiography at six months confirmed the presence of the sternal wire within the ascending thoracic aorta (Figure 2).

After multidisciplinary consultation with cardiovascular and thoracic surgery teams, surgical removal of the sternal wire was planned. The patient remained asymptomatic, so surgery was scheduled after completing adjuvant treatment.

Fifteen months after the initial surgery, removal of the sternal wire from the ascending thoracic aorta was performed. The procedure included partial sternotomy with an oscillating saw, wire transection close to the aortic wall, and extraction along the wire’s axis. There was no significant surrounding hematoma and no evidence of active bleeding at the time of inspection. The steel wire appeared to be intra-aortic but with a probable gradual indentation of the aortic wall. Extracorporeal circulation was prepared as a backup but was not required. There was no bleeding during the procedure, and the sternum was closed conventionally with three new sternal wires.

**Figure 3:**
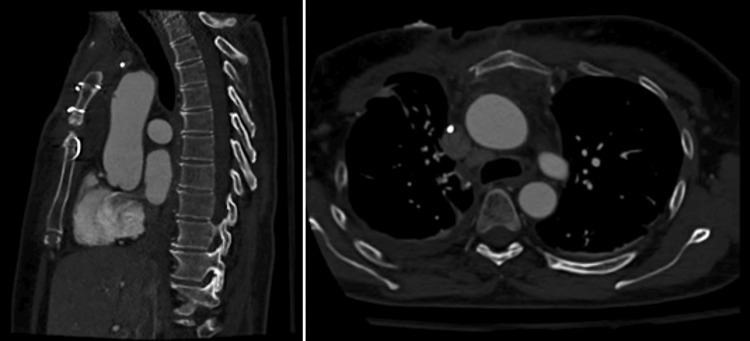
Postoperative computed tomography angiography after removal of the sternal wire

Postoperative computed tomography angiography performed on day two showed no local complications (Figure 3). Clinical and radiographic follow-up at two weeks post-surgery was satisfactory.

## Discussion

Only a small number of case reports in the literature describe injuries to major anterior mediastinal structures associated with sternal wires, most of which involve fractured wires. Several publications have reported migration of these fractured wires into critical structures such as the ascending aorta, right ventricle, pulmonary artery, or bronchus. Recent reviews by Mokhtar et al. (2020) and Gallego-Navarro et al. (2024) have summarized these rare but serious events [2,3]. Mokhtar et al. specifically highlighted the value of computed tomography angiography in cases where standard chest radiography fails to clearly determine the wire’s relationship to adjacent mediastinal structures [2]. Although rare, cardiovascular injuries caused by retained foreign bodies, such as sternal wires, remain potentially life-threatening complications [4].

Aortic wall injury resulting from unfractured sternal wires is an exceptionally rare complication. Müller and Ferencz described a case involving the development of a pseudoaneurysm of the ascending aorta that required surgical intervention [5]. In contrast, our patient presented a distinct scenario in which the unfractured sternal wire gradually penetrated the aortic wall without causing pseudoaneurysm formation or clinical symptoms, and was discovered incidentally during routine imaging follow-up. The absence of hemorrhage during wire removal suggests the wire had become incorporated into the wall of the ascending thoracic aorta as a foreign body. Although computed tomography angiography images demonstrated an apparent intraluminal position, the wire was not truly intraluminal. We propose to describe this unusual presentation as a form of "mechanical endocytosis", by analogy to the cellular process in which foreign material is gradually internalized.

Most cases described in the literature presented with hemodynamic instability, often secondary to cardiac tamponade from injuries to the ascending aorta or right ventricle [4,6-8]. Surgical repair in these scenarios is frequently performed under extracorporeal circulation [9,10]. Consequently, it is essential to have cardiopulmonary bypass readily available and a cardiothoracic surgical team prepared for emergent intervention. In the present case, a multidisciplinary plan was coordinated in advance with our cardiothoracic surgery department.

Additionally, many reported cases involved patients with a history of pectus excavatum repair [4,6,7,10,11], a correlation also highlighted by Mieno et al. [4]. This may be attributable to the reduced anteroposterior thoracic diameter in such patients, which increases the likelihood of direct contact between the posterior sternal surface and adjacent mediastinal structures, including the ascending aorta.

Finally, we believe that suboptimal sternal closure contributed to the pathogenesis in this case. Proper tightening of sternal wires is critical to prevent wire loosening or posterior displacement. This issue was also illustrated by Müller and Ferencz, who demonstrated a loose wire on a lateral chest X-ray [5]. Such loosening may have facilitated the progressive incorporation-or "mechanical endocytosis"-of the wire into a major mediastinal structure.

Note: The term "mechanical endocytosis" is used metaphorically to describe the gradual incorporation of a foreign body into a vascular structure, in the absence of acute trauma, bleeding, or pseudoaneurysm.

## Conclusions

This case report presents an exceptional computed tomography angiography image showing an unfractured sternal wire penetrating the ascending thoracic aorta, a rare and, to our knowledge, previously unreported complication. Unlike most documented cases involving fractured wires and presenting with cardiac tamponade or pseudoaneurysm, this patient remained asymptomatic. We propose the term “mechanical endocytosis” to describe this unique mechanism of gradual wire integration into the aortic wall. This case underscores the critical importance of precise wire placement and secure tightening during sternal closure in both cardiac and thoracic surgery. Early detection through imaging and a multidisciplinary surgical approach enabled safe management without the need for extracorporeal circulation. Meticulous technique and postoperative vigilance remain essential to preventing such potentially life-threatening complications.
